# Multiscale biphasic modelling of peritumoural collagen microstructure: The effect of tumour growth on permeability and fluid flow

**DOI:** 10.1371/journal.pone.0184511

**Published:** 2017-09-13

**Authors:** Peter A. Wijeratne, John H. Hipwell, David J. Hawkes, Triantafyllos Stylianopoulos, Vasileios Vavourakis

**Affiliations:** 1 Department of Computer Science, University College London, London, United Kingdom; 2 Department of Medical Physics and Biomedical Engineering, University College London, London, United Kingdom; 3 Department of Mechanical and Manufacturing Engineering, University of Cyprus, Nicosia, Cyprus; University of Michigan, UNITED STATES

## Abstract

We present an in-silico model of avascular poroelastic tumour growth coupled with a multiscale biphasic description of the tumour–host environment. The model is specified to in-vitro data, facilitating biophysically realistic simulations of tumour spheroid growth into a dense collagen hydrogel. We use the model to first confirm that passive mechanical remodelling of collagen fibres at the tumour boundary is driven by solid stress, and not fluid pressure. The model is then used to demonstrate the influence of collagen microstructure on peritumoural permeability and interstitial fluid flow. Our model suggests that at the tumour periphery, remodelling causes the peritumoural stroma to become more permeable in the circumferential than radial direction, and the interstitial fluid velocity is found to be dependent on initial collagen alignment. Finally we show that solid stresses are negatively correlated with peritumoural permeability, and positively correlated with interstitial fluid velocity. These results point to a heterogeneous, microstructure-dependent force environment at the tumour–peritumoural stroma interface.

## Introduction

The importance of mechanics in cancerous growth, invasion and metastasis is well-established ([1], [2]). In terms of cancer mechanobiology, of particular interest is how interactions between a tumour and its host tissue influence its behaviour [3]. These interactions occur across multiple length and time scales, and include—among other factors—solid stress generation induced by host tissue displaced during growth, collagen remodelling in the extracellular matrix (ECM) at the tumour periphery, and interstitial fluid flow between the tumour and the host tissue. Recent observations have shown that microstructural properties of the host tissue—such as collagen fibre density, alignment and cross-link density—are instrumental in the development and progression of solid tumours [4] and are subject to remodelling during tumour progression [5]. Similarly, interstitial fluid flow is induced and altered by both the growth itself and the associated tumour-host interactions [6], [7].

Given the potentially correlated nature of these observations, it is instructive to build a reductionist mathematical model with which to isolate and test each component separately. In order to make such a model biologically relevant, it should be parametrised by measurable biophysical properties at multiple scales. This motivates the development of a multiscale, multiphase model that can be coupled with a model of tumour growth. A brief overview of pertinent models is given here; see e.g. [8], [9] for comprehensive reviews of mathematical tumour modelling.

Pioneering work by [10] investigated the role of the ECM in interstitial fluid transport in tumours using a biphasic continuum model. A poroelastic continuum model was proposed by [11], specified to experimental data and used to test the effect of solid stress on growth. Mixture modelling was utilised by [12] to develop a thermodynamically-consistent continuum model of multispecies tumour growth. This model was then used to investigate tumour invasion, morphology and angiogenesis [13]. More recently, a review of competing continuum models was performed [14] and judged a multiphase flow model in a deformable porous ECM to require the least number of assumptions in its derivation, and have the most potential for development. A similar model was employed by [15], [16], with an adapted formulation that accounted for residual stress. This model was used to recapitulate observed effects of solid stress on tumour growth, and showed that both cells and blood vessels can be compressed by growth-induced stress.

To our knowledge there exists no multiple length scale description of biphasic tumour growth. The model we present here uses the biphasic formulation proposed by [17] to extend our previous work [18], which was solely defined in terms of solid mechanics. Therefore while the individual components of the model are not new, their coupling is, providing a novel computational model of avascular tumour growth with which we can perform biologically relevant hypothesis tests.

Here we conduct simulations of in-vitro tumour spheroid growth into a collagen hydrogel to test that i) tumour growth causes passive microstructural remodelling; ii) collagen microstructure influences macroscale fluid flow; and iii) solid stress is correlated with tissue permeability and interstitial fluid flow. The paper is structured as follows: ‘Material and methods’ provides a description of multiscale tumour modelling and its numerical implementation, including a link to our open-source software; ‘Results’ presents the set of hypothesis tests using the proposed model; and ‘Discussion’ critiques the results and methods.

## Materials and methods

### Mathematical model

**Fig 1:**
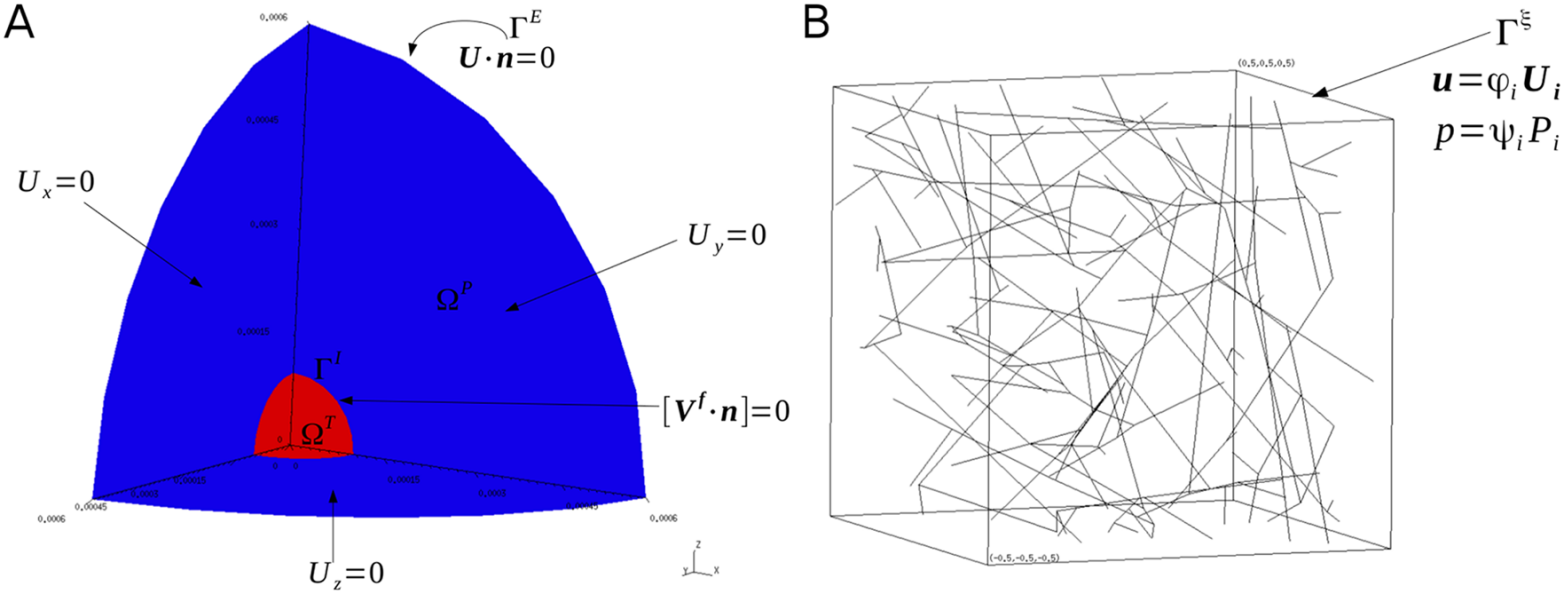
**A: Schematic representation of the macroscopic domain**. From inside out: tumour (Ω^*T*^), tumour-peritumoural stroma interface (Γ^*I*^), peritumoural stroma (Ω^*P*^), and external boundary (Γ^*E*^). The symmetry surfaces referenced in the text (Γ^*S*^) are the set of boundaries with fixed displacements. **B: Schematic representation of an example representative volume element (RVE) from the microscopic domain.** The RVE domain is denoted by *ω*, and its outer surfaces are labelled *ξ*. Proper boundary conditions are shown in each figure.

The domain of interest, defined in Fig 1, is comprised of a spherical tumour, Ω^*T*^, embedded in a shell of peritumoural stroma, Ω^*P*^, with a boundary interface between the tumour and peritumoural stroma, Γ^*I*^, and an external boundary, Γ^*E*^. For simplification, spherical symmetry is assumed and only one eighth of the total domain is analysed; symmetry surfaces are collectively denoted by Γ^*S*^. The tumour is defined at the macroscopic scale only, while the peritumoural stroma (PTS) is treated as a multiscale material. As with our previous work [18], and following [19], volume averaging theory is used to couple the micro and macroscopic scales. The macroscale is described as a poroelastic continuum, and the microscale is discretised as a set of three-dimensional cubic domains, *ω*, with surfaces *ξ*, termed representative volume elements (RVEs). Each RVE contains a hydrated collagen fibre network, represented by a scaffold of one-dimensional incompressible trusses connected at pin joints which permit translations and rotations, thus mimicking the behaviour of cross-linked collagen fibres. The models and their coupling are described in the following sections.

#### Macroscale model

A mixed *U* − *P* formulation is utilised at the macroscale [20]. The conservation of linear momentum in a poroelastic continuum, assuming zero inertia and viscosity, is expressed in a Lagrangian framework as:

$$\begin{array}{c} \nabla \cdot (F\cdot S-P)-Q=0 \end{array}$$ (1)

Where $F=I+\frac{\partial U}{\partial X}$ is the deformation gradient with respect to the initial configuration, ***S*** is the 2nd Piola-Kirchoff stress tensor, *P* is the fluid pressure and vector ***Q*** contains body forces and source terms (defined later). Throughout we use $\nabla \equiv \frac{\partial }{\partial X}$, where ***U*** and ***X*** are the displacement and position vectors in the Lagrangian frame of reference. Note that the reference configuration is assumed undeformed and hence initial stresses are zero.

The conservation of mass in the medium is expressed as:

$$\begin{array}{c} \frac{1}{M}\frac{\partial P}{\partial t}+\theta^{S}\nabla \cdot V^{S}+\theta^{F}\nabla \cdot V^{F}-Q^{F}=0 \end{array}$$ (2)

Where ***V***^*S*(*F*)^ are the solid (fluid) velocities and *Q*^*F*^ are body forces and source terms (defined later). The term $\frac{1}{M}$ is the Biot storage coefficient; here we define *M* = *κ*_*u*_, the undrained solid bulk modulus [21]. Finally, *θ*^*S*(*F*)^ are the solid (fluid) volume fractions, which obey conservation:

$$\begin{array}{c} \theta^{S}+\theta^{F}=1 \end{array}$$ (3)

To solve the forward problems for displacement, ***U***, and pressure, *P*, it is necessary to define constitutive equations. In the tumour domain, Ω^*T*^, a hyperelastic strain-energy density function, *W*, is used to described the solid mechanics [22]:

$$\begin{array}{c} W=\frac{\mu }{2}\;\left(I_{1}-3\right)+\frac{\kappa }{2}\;{\left(J-1\right)}^{2} \end{array}$$ (4)

Where for small deformations the material coefficients *μ* and *κ* correspond to the drained shear and bulk moduli, respectively, while invariant $I_{1}=\text{tr}\left(F_{e}^{T}F_{e}\right)=\text{tr}\left(C_{e}\right)$, and determinant *J* = det(***F***_*e*_). To describe growth, we multiplicatively decompose the deformation gradient, ***F***, into elastic, ***F***_*e*_, and inelastic (growth), ***F***_*g*_, components: ***F*** = ***F***_*e*_ ⋅ ***F***_*g*_ [18]. The corresponding 2nd Piola-Kirchoff stress can then be obtained from the relation $S=2\;\frac{\partial W}{\partial C_{e}}$. In the PTS domain, Ω^*P*^, a multiscale constitutive description is used (see sub-section ‘Scale coupling’).

Following [17], a multiscale form of Darcy’s law is used to describe the fluid mechanics in both domains:

$$\begin{array}{c} V^{F}=-K\cdot \left(\theta^{F}\nabla P+d\right) \end{array}$$ (5)

Where ***K*** is the positive definite conductivity matrix and ***d*** is a source term arising from the microscale formulation. Here ***K*** = *k*_*o*_ ***I*** and ***d*** = 0 in Ω^*T*^, and are derived from the microscale model in Ω^*P*^ (see sub-section ‘Scale coupling’).

The macroscale model is completed with the following initial and boundary conditions:

$$\begin{array}{c} \begin{array}{c} U=0,\;t=0\\ {}[S\cdot \hat{n}]=0,X\in \Gamma^{I}\\ U\cdot \hat{n}=0,X\in \Gamma^{S,E} \end{array}\}\text{solid}\\ \begin{array}{c} P=0,\;t=0\\ {}[V^{F}\cdot \hat{n}]=0,X\in \Gamma^{I,S,E} \end{array}\}\text{fluid} \end{array}$$ (6)

Here Γ^*I*,*E*^ are interface and external boundaries (see Fig 1A), Γ^*S*^ are the symmetry surfaces (corresponding to the axial planes in Fig 1A), [⋅] denotes the change across a boundary (i.e. $\left[S\cdot \hat{n}\right]\equiv S_{1}\cdot \hat{n}=S_{2}\cdot \hat{n}$, where 1, 2 correspond to contiguous domains), and $\hat{n}$ is the outward facing unit vector on a given surface.

Finally, the growth-induced deformation in Ω^*T*^—and hence the progression of interface Γ^*I*^—is introduced through the following expression of isotropic tumour growth:

$$\begin{array}{c} F_{g}=f\left(t\right)I \end{array}$$ (7)

Where *f*(*t*) can be any smooth function in time, *t*. Here a Gompertz-type expression of the form *f*(*t*) = *α* exp(−*β* exp(−*γt*)) is used [18].

#### Microscale model

At the microscale, the boundary value problem is established by interpolating the macroscale displacement to the RVE boundary nodes, which are defined in parametric (i.e. dimensionless) space. The balance of linear momentum with respect to the reference configuration is then solved at each pin joint in each microscale mesh:

$$\begin{array}{c} \nabla \cdot (F^{f}\cdot s-p)=0 \end{array}$$ (8)

Here $F^{f}=I+\frac{\partial u}{\partial x}$ is the fibre deformation gradient, ***s*** is the microscale 2nd Piola-Kirchoff stress tensor and *p* is the microscale fluid pressure. As in [18], the collagen fibre constitutive equation assumes an exponential form with a negative phase to describe fibre compression:

$$\begin{array}{c} f=\frac{a^{f}e^{f}}{c_{0}}\;\left[\text{exp}\left(c_{0}\epsilon \right)-1\right]\;{\hat{n}}^{f} \end{array}$$ (9)

Here ***f*** is the axial force along the fibre, *a*^*f*^ is the fibre cross-sectional area, *e*^*f*^ is the fibre stiffness, *c*_0_ is a material constant, *ϵ* is the Green strain and ${\hat{n}}^{f}$ is the fibre unit vector. Hence ***s*** is given by ***s*** = (***F***^*f*^)^−1^ **⋅** ***f***/*a*^*f*^, where *a*^*f*^ is the fibre undeformed cross-sectional area. Unlike the macroscale, no equation is solved for the fluid pressure; instead, it is interpolated directly from the macroscale solution (see sub-section ‘Solution method’). Body forces are assumed zero.

The following initial and boundary conditions complete the microscale model:

$$\begin{array}{c} \begin{array}{c} u=0,\;t=0\\ u=\phi_{i}U_{i},x\in \Gamma^{\xi } \end{array}\}\text{solid}\\ \begin{array}{c} p=0,\;t=0\\ p=\psi_{i}P_{i},x\in \omega \end{array}\}\text{fluid} \end{array}$$ (10)

The boundary displacements and the pressure are interpolated from the respective macroscopic solutions using the quadratic and linear Lagrange polynomials *ϕ*_*i*_, and *ψ*_*i*_, respectively. This process is explained in sub-section ‘Solution method’.

#### Scale coupling

Using volume averaging theory [23], the macroscopic field can be defined as a volume average of the microscopic fields on the RVE surface:

$$\begin{array}{c} S=\frac{1}{V}\int_{\xi }s\;d\omega \end{array}$$ (11)

Here *V* is the RVE volume in the three-dimensional parametric space, ***S*** the macroscopic stresses (as defined in sub-section ‘Macroscale model’) and $s={\hat{n}}^{f}⊗\hat{n}\cdot f$ the microscale stresses, where $\hat{n}$ is the outward surface normal. In principal any statistically homogeneous field is viable. Accordingly, and following [19] and [17] respectively, the equivalent expressions for the macroscopic source term ***Q*** and permeability ***K*** are:

$$\begin{array}{c} Q=\frac{1}{V}\int_{\xi }\left(s-S\right)\cdot u^{\prime }\cdot \hat{n}\;d\omega \end{array}$$ (12)

Here ***u***′ is the directional derivative of the microscale displacement, $u^{\prime }=\frac{\partial u}{\partial x}{|}_{x\in \xi }$. Similarly:

$$\begin{array}{c} K=\frac{1}{V}\sum_{z}k \end{array}$$ (13)

Here the sum is over all fibres, *z*, and ***k*** is the fibre permeability, defined as ***k*** = (***r***^*T*^ ⋅ ***c*** ⋅ ***r***)^−1^, where ***r*** is the fibre direction cosine matrix (i.e. the mapping between the fibre and fluid frames) and ***c*** is the diagonalised fibre drag coefficient matrix. The diagonal components are set equal to expressions previously derived for parallel [24] and perpendicular [25] steady flow in a square array of cylinders:

$$\begin{array}{ccc} c_{11} & = & \frac{4\pi \ell }{-\text{ln}\left(\theta^{S}\right)-1.476+2\theta^{S}-0.5{\left(\theta^{S}\right)}^{2}}\\ c_{22} & = & c_{33}=\frac{8\pi \ell }{-\text{ln}\left(\theta^{S}\right)-1.476+2\theta^{S}-1.774{\left(\theta^{S}\right)}^{2}} \end{array}$$ (14)

Here *ℓ* is the fibre length in the current frame. As such, for low solid volume fractions (*θ*^*S*^ → 0), *c*_22,33_ ≈ 2*c*_11_. The use of these expressions was justified by [26], who made comparisons between RVEs of this type and CFD simulations. Following [17], the scale mixing term *d* is defined as:

$$\begin{array}{c} d=\frac{1}{V}\sum_{z}k\cdot v^{S} \end{array}$$ (15)

Here ***v***^*S*^ is the displacement rate of the microscale solid phase. Finally, and again following [17], the fluid source term *Q*^*F*^ is given by:

$$\begin{array}{c} Q^{F}=\frac{a^{f}}{V}\sum_{z}\left(V^{S}-v^{S}\right)\cdot \hat{n} \end{array}$$ (16)

Given that the mathematical formulation requires the RVE to be defined in parametric space, a dimensionalisation term is necessary to convert the upscaled parameters to physical space. Following [19], this assumes the form:

$$\begin{array}{c} \eta =\sqrt{\frac{\theta^{S}V}{a^{f}L}} \end{array}$$

Here *L* is the length sum of all fibres in the RVE. Substituting ***x***^*dim*^ = *η* ***x*** into the above equations yields the dimensionalised variables (e.g. *dω*^*dim*^ = *η*^3^ *dω*; (***v***^*S*^)^*dim*^ = *η* ***v***^*S*^; and so on).

### Solution method

The weak forms of Eqs (1) and (2) are discretised using the Galerkin finite element (FE) method [27] with Lagrange polynomials, and solved using an explicit Total Lagrangian method, whereas at the microscale, Eq (8) is solved using an implicit Total Lagrangian method. See S1 File for the full set of FE equations.

The algorithmic structure is similar to that detailed in our previous work [18], but extended to incorporate the multiscale fluid phase. In brief, the following steps are performed:

1. Macroscopic boundary value problem: solve Eqs (1) and (2) for ***U*** and *P*, respectively, using a Total Lagrangian (TL) explicit solver.
2. Downscale: interpolate macroscale nodal ***U*** solutions to RVE boundary nodes, and the *P* solutions to every RVE node. This is done by interpolation using Lagrange polynomials: ***u*** = *ϕ*_*i*_***U***_*i*_ and *p* = *ψ*_*i*_*P*_*i*_, where *ϕ*_*i*_, *ψ*_*i*_ are the interpolation functions and the sum is over all nodes in the macroscopic FE.
3. Microscopic boundary value problem: apply the displacements prescribed by the downscaling and solve Eq (8) for ***u*** using a TL implicit solver.
4. Upscale: use Eqs (11), (12), (13), (15) and (16) to calculate the volume averaged parameters ***S***, ***Q***, ***K***, ***d*** and *Q*^*F*^, respectively.

These steps are repeated until the simulation reaches the desired end time point. The algorithm itself is implemented in C++ in an open-source, parallelised finite element solver framework developed by the authors (FEB3: Finite Element Bioengineering in 3D, available upon request from https://bitbucket.org/vasvav/feb3-finite-element-bioengineering-in-3d/wiki/Home). The framework incorporates various open-source scientific computing libraries, specifically: libMesh [28], PETSc [29], blitz++, GSL and MPICH.

**Fig 2:**
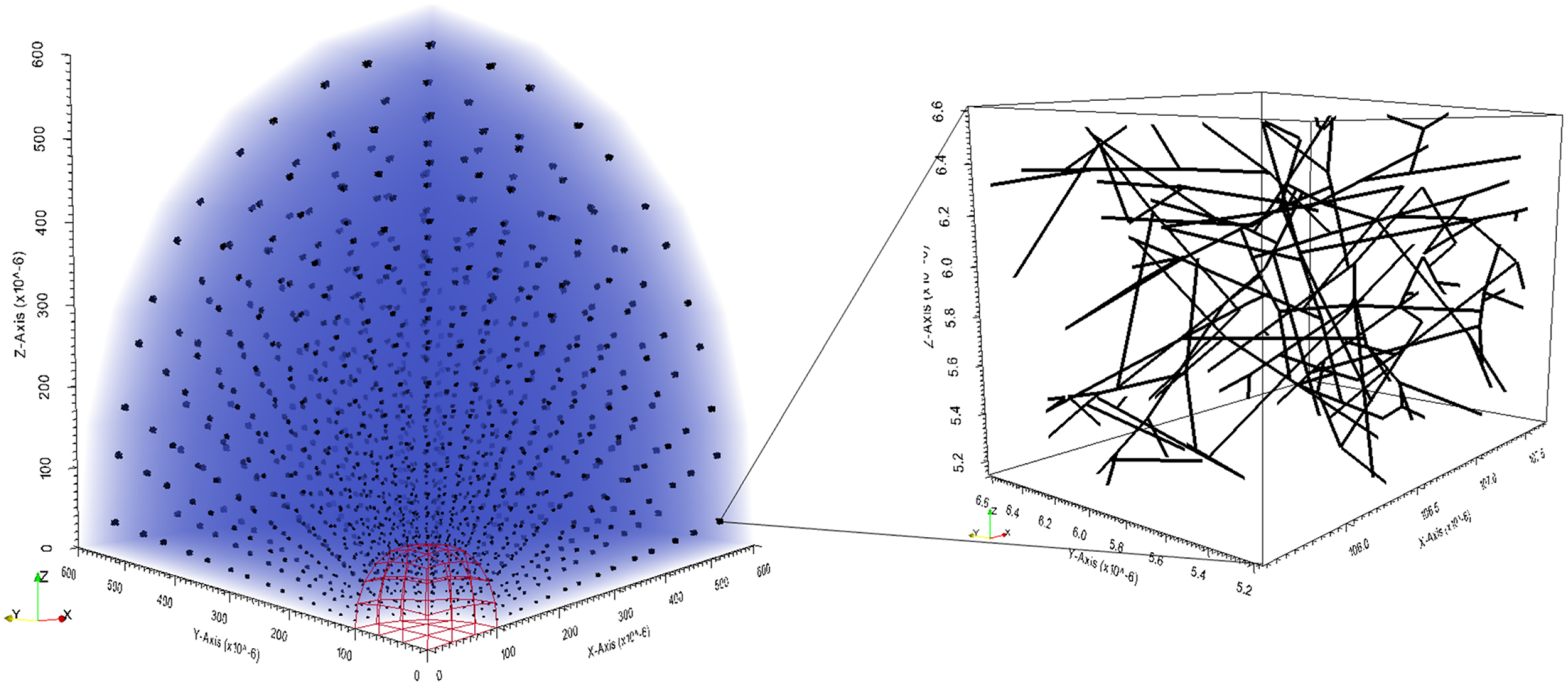
Initial state meshes at both scales. The tumour spheroid octant is shown as a red wire frame and has radius 0.1 mm; the peritumoural stroma is shown as an opaque blue volume has thickness 0.5 mm; and each RVE has a side length of approximately 20 microns (axes are not to scale).

The open-source finite element mesh generator Gmsh was employed to produce meshes at the macroscopic scale. The mesh used for the primary simulations is shown in Fig 2: it comprised of 216 hexahedral elements at the macroscale and 326,592 truss elements at the microscale, corresponding to 323 nodes at the macroscale and 308,448 nodes at the microscale, respectively. Each collagen fibre network was generated separately, resulting in RVEs with random orientation, total fibre length and number of cross-links. The number of elements per RVE ranged between [200, 300], which has previously been shown to render the average stress independent of the total number of elements [19]. Eight- and two-point Gauss-Legendre quadrature rules were used for the solid mechanics at the macro- and micro-scales, respectively, and a single quadrature point for the fluid mechanics. This reduced order integration was chosen to avoid volumetric locking whilst maintaining a computationally tractable solution time.

## Results

Values of the model parameters used in the following simulations are provided in S1 Table. The material properties were chosen to simulate an in-vitro tumour spheroid with radius 0.1mm growing into a dense hydrated collagen gel with thickness 0.5mm; the latter was chosen to ensure boundary effects were negligible. The tumour was allowed to grow to equilibrium, which for the chosen collagen stiffness and volume fraction resulted in an increase in radius of ∼25%, corresponding to a total peritumoural stroma (PTS) strain of ∼5%. While this is a relatively small total increase, it is in agreement with previously reported experiments of tumour spheroid growth into stiff surroundings [30].

### Tumour growth causes passive microstructural remodelling

**Fig 3:**
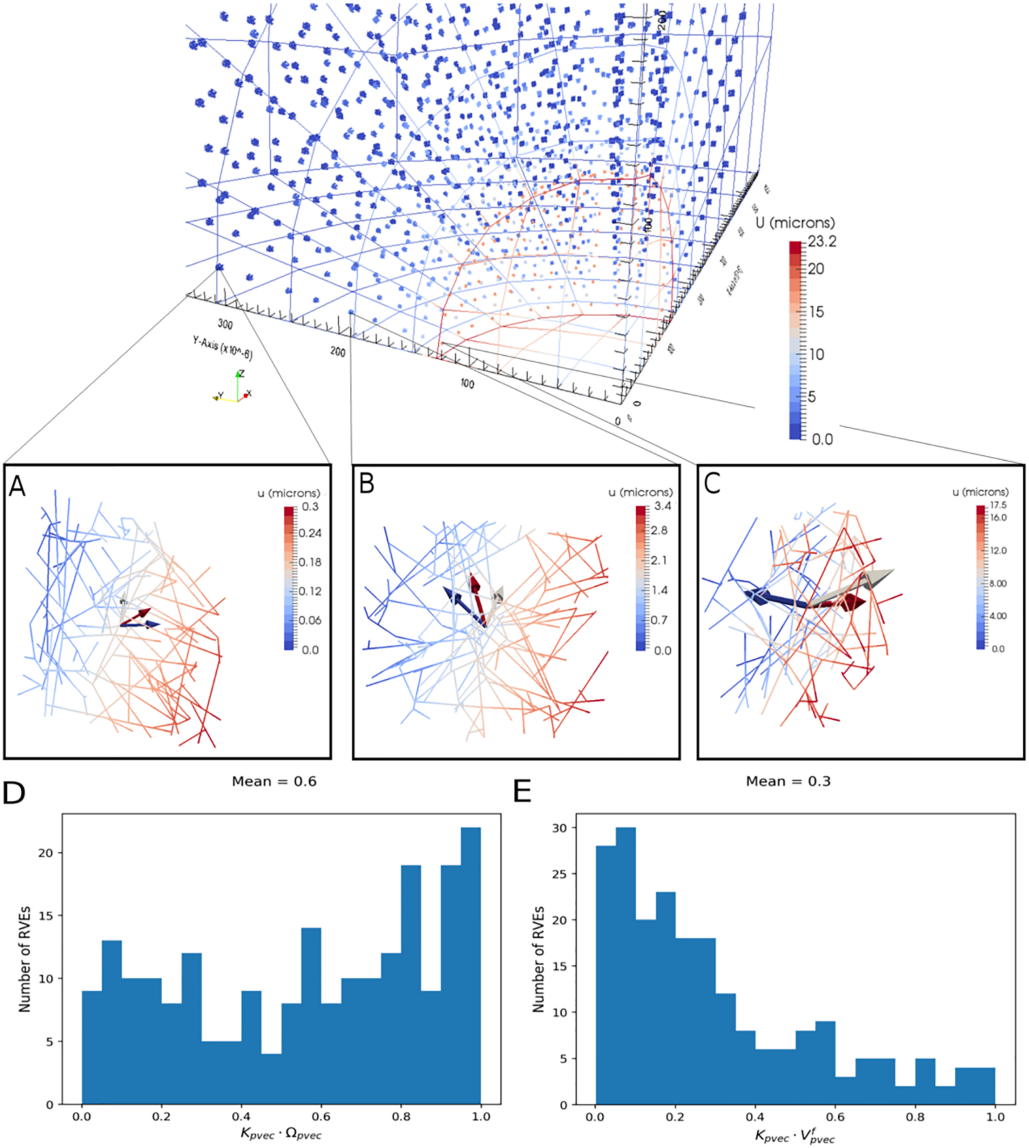
Tumour growth causes collagen remodelling. From top to bottom: final state peritumoural stroma mesh (both scales); final state RVEs from the (A) 5th, (B) 3rd and (C) 1st layers of the PTS; (D) histograms of the scalar product between the principal network permeability, *K*_*pvec*_, and principal network orientation vectors, Ω_*pvec*_; and (E) the scalar product between the principal network permeability and interstitial fluid velocity, *V*_*pvec*_, for each RVE in the 1st layer of elements in the PTS. The arrows centred on each RVE show the principal eigenvectors of the network orientation (grey) and permeability (red), and the interstitial fluid velocity (blue).

Final state displacements are shown in Fig 3 (top) at both scales. A high level of network compression is observed in the near-field, with deformations dropping to zero approximately halfway into the PTS. This is in agreement with our previous findings [18], which showed that compression was mainly localised to the tumour-PTS boundary. Also in agreement with both our previous findings and experimental observations by others [4] are the network alignments at the boundary: passive mechanical remodelling causes a shift from an initially random to a circumferential alignment with respect to the tumour boundary. The principal network orientation is calculated from the average RVE orientation tensor, Ω_*ij*_, defined as [31]:

$$\begin{array}{c} \Omega_{ij}=\frac{\sum (\ell_{i}\cdot \ell_{j})/\ell }{\sum \ell } \end{array}$$

**Fig 4:**
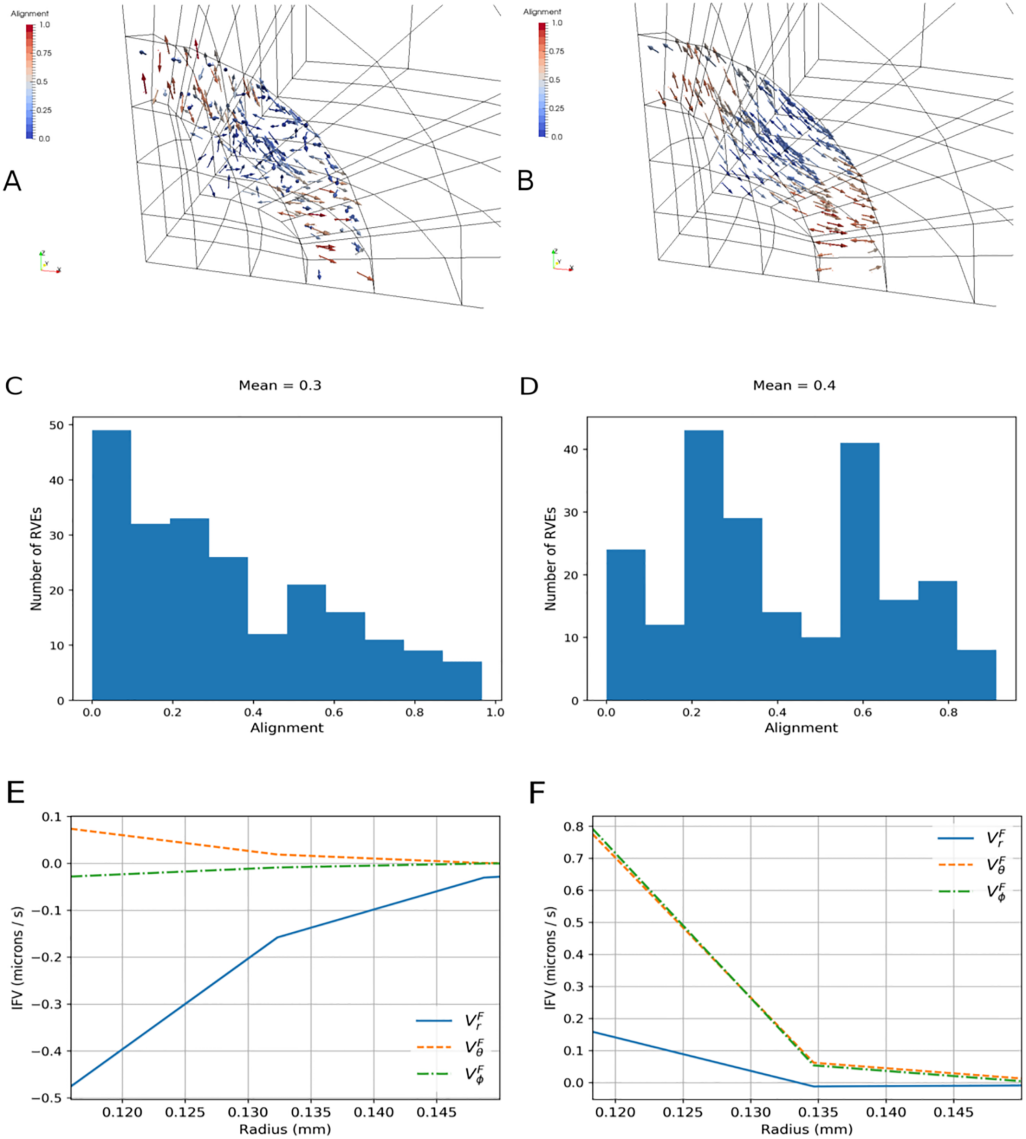
Initial collagen microarchitecture influences final collagen alignment and fluid flow. Row-wise from top: Final state network principal orientation eigenvectors in the first layer of the PTS, for an initially (A) random and (B) aligned PTS. The colour bar indicates alignment with respect to the tumour boundary: 0 for circumferential, 1 for perpendicular. Final state network alignments for an initially (C) random and (D) aligned PTS. Final spherical IFV components near the tumour boundary, for an initially (E) random and (F) aligned PTS.

Here *ℓ*_*i*_ is the projection of a fibre of length *ℓ* in the *i*-direction, and the sum is over all fibres in the RVE. Random networks are approximately isotropic; hence Ω_11_ ≈ Ω_22_ ≈ Ω_33_ = 1/3. The scalar product of the principal eigenvector of Ω_*ij*_ and the outward facing normal of the finite element to which the RVE belongs gives the alignment with respect to the boundary: equal to 1 if perpendicular and 0 if parallel. A final state contour map of principal RVE orientations and a histogram of their alignments in the first layer of the PTS are shown in Fig 4A and 4C, respectively. Given that our previous model was solely defined in terms of solid mechanics, the similarity between [18] and Fig 4C suggests that fluid flow does not play a role in this type of remodelling.

### Collagen microstructure influences macroscale fluid flow

To demonstrate the capabilities of the multiscale biphasic model, example hydrated collagen networks are selected from the first, third and fifth layer of the PTS and shown in Fig 3A-3C, respectively. Each figure shows the deformed network, corresponding nodal displacements, and three vectors: the principal network orientation (grey), principal network permeability (red) and interstitial fluid velocity (IFV) direction (blue). The principal network permeability is equal to the principal eigenvector of the permeability tensor, ***K*** (Eq (13)), and the IFV is calculated from Eq (5). A number of interesting features are predicted:

- The angle between the principal orientation and permeability vectors is generally acute. This is supported by Fig 3D, which shows a histogram of the scalar product between the principal network permeability and principal network orientation vectors in each RVE from the first layer of finite elements in the PTS; its mean is greater than 0.5. These results reflect the constitutive description (Eq (14)), which assumes that fluid flows more easily parallel rather than perpendicular to fibres.
- The IFV points approximately perpendicular to the permeability in all networks. This is supported by Fig 3E, which shows a histogram of the scalar product between the principal network permeability and IFV vectors in each RVE from the first layer of finite elements in the PTS; its mean is less than 0.5. This is a result of radial tumour growth, which produces a radial gradient of the interstitial fluid pressure (IFP).
- The angle between the IFV and permeability closes as we move radially out. This indicates that the IFP gradient drives the IFV direction at the boundary, but further out the permeability begins to direct the flow.

To further investigate the effect of collagen microstructure on peritumoural IFV, simulations were compared between networks with the default (i.e. random) initial orientation and prealigned networks, whereby every network was given the same structure and hence orientation: Ω_11_ ≈ 0.39, Ω_22_ ≈ 0.23, Ω_33_ ≈ 0.38. The resulting alignment contours and histograms are depicted in Fig 4A-4D. As previously noted, the initially random networks realign circumferentially and the growth produces a dominantly radial IVF at the boundary (Fig 4E). In contrast, the prealigned networks remodel into a mix of parallel and perpendicular alignments. This causes a more irregular tumour morphology, which drives up the circumferential IFP gradients and hence the magnitude of the circumferential IFV (Fig 4F). Comparing Fig 4E and 4F suggests a greater dependency of IFV on the initial orientation of the microstructure than the final; despite the greater number of final-state circumferentially aligned networks in the default simulation, the circumferential IFV is larger in the prealigned simulation because of the more anisotropic tissue deformation.

**Fig 5:**
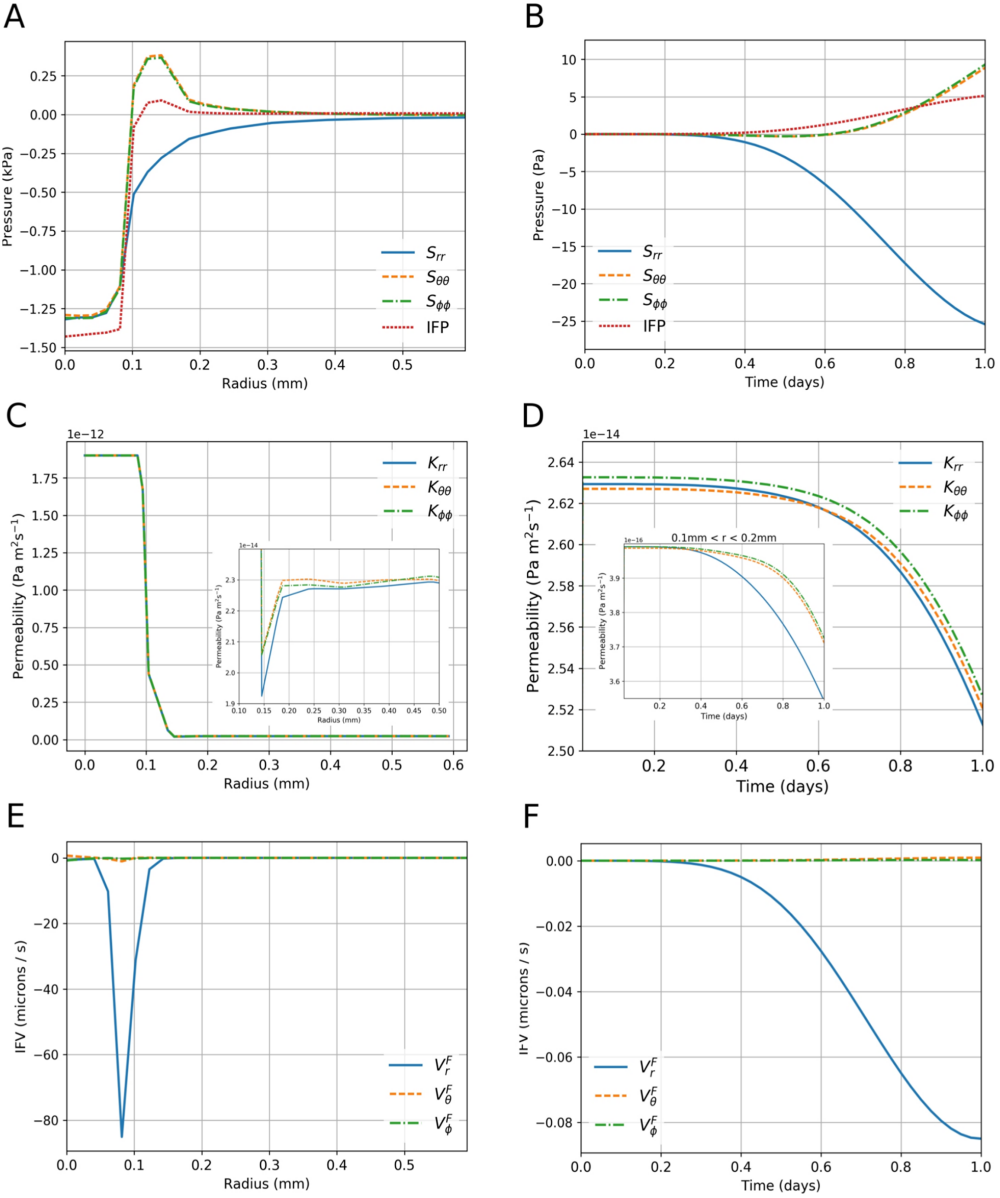
Final state variables in space and time. A: Final spherical solid stress components and IFP over radial distance. B: Final spherical solid stress components and IFP over time, integrated over the PTS volume. C: Final spherical permeability components over radial distance; inset shows a zoom into the tumour-PTS boundary. D: Final spherical permeability components over time, integrated over the PTS volume; inset shows the same for the region 0.1mm <*r* < 0.2mm. E: Final spherical IFV components over radial distance. F: Final spherical IFV components over time, integrated over the PTS volume. All time-dependent plots are normalised with respect to the initial volume of the PTS.

To facilitate comparison with experimental data, the macroscale solid stress, IFP, permeability and IFV are plotted against space and time in Fig 5 for the simulation into the random PTS. In the plots with respect to time, variables are integrated over the PTS domain only in order to focus on peritumoural properties.

- Fig 5A: as expected, compressive solid stresses are observed inside the tumour. At the tumour boundary the radial component remains negative, while the circumferential components become positive: this reflects the stretching of collagen fibres around the tumour periphery. All components drop to zero in the far field. These predictions support the notion of a heterogeneous force environment at the boundary, and are consistent with previous studies of growth-induced solid stress [15], [32]. An approximately flat IFP is observed inside the tumour, followed by a strongly positive gradient near the boundary. This is in qualitative agreement with past observations (Fig 2 in [11]; Fig 4A in [33]) and demonstrates that, during avascular tumour development, IFP in the PTS is driven by tumour growth: it is elevated at the boundary, where the tissue is most compressed, before falling off and reaching a plateau away from the tumour.
- Fig 5B: radial solid stress increases negatively with time in the PTS, while circumferential stresses increase positively. This is due to radial compression and circumferential extension of the fibre networks. IFP increases positively and, as with the solid stresses, follows the shape of tumour growth.
- Fig 5C: all permeability components decrease with increasing radius, with approximately two orders of magnitude difference between the tumour and PTS. To examine the effect of collagen remodelling, the inset plot shows a zoom into the boundary. A sharp decrease in all permeability components is predicted at the interface due to network compression. Furthermore, a greater decrease is predicted in the radial than circumferential components, reflecting the reorientation of collagen to a circumferential alignment with respect to the tumour boundary. All components converge to approximately the same value further from the boundary.
- Fig 5D: the radial component of permeability decreases more rapidly with time than the circumferential components. This effect is strongest at the boundary; see the inset plot, which shows the permeability integrated over a region at the tumour-PTS boundary (0.1mm <*r* < 0.2mm). As with Fig 5C, this is driven by the passive network remodelling at the boundary: collagen is remodelled circumferentially at the boundary, making the networks more permeable in the circumferential direction.
- Fig 5E: IFV is approximately a factor of 100 greater at the boundary than inside or outside the tumour, and it points inwards. This demonstrates that fluid flows from the PTS inside the tumour as a result of the tumour being more permeable. The magnitude of the IFV throughout the PTS is in qualitative agreement with experimental measures of interstitial fluid flow (0.6 ± 0.2*μ*m/s [34]).
- Fig 5F: the radial component of the IFV is much larger than the circumferential components. This is due to circumferential deformation being only slightly anisotropic; as the initial distribution of network orientations is approximately flat, the growth produces an approximately spherical morphology, and hence the circumferential pressure gradients are small.

### Solid stress is correlated with permeability and interstitial fluid velocity

**Fig 6:**
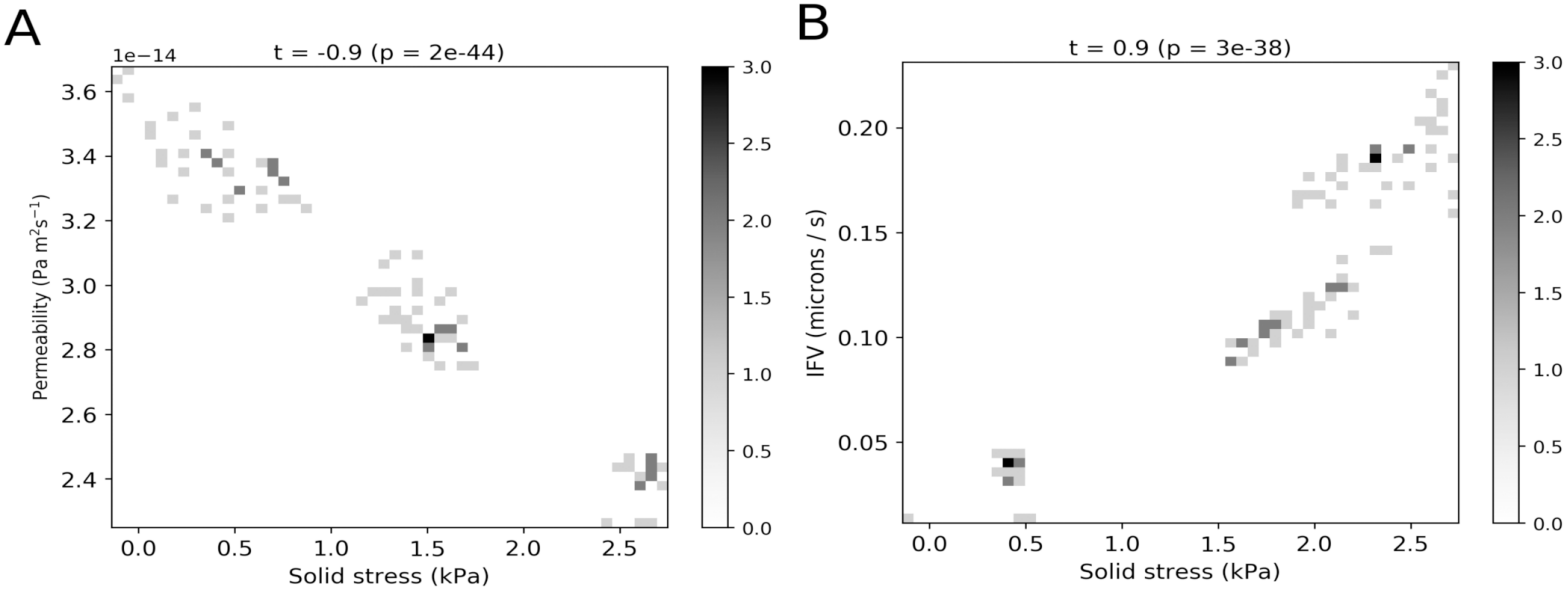
Solid stress, permeability and fluid velocity correlations. A: Two dimensional histogram of final state solid stress versus permeability. B: Two dimensional histogram of final state solid stress versus interstitial fluid velocity (IFV). The Spearman rank correlation coefficient, t, and its corresponding p-value, p, are shown at the top of each plot. Statistical tests were performed using the SciPy stats module (https://www.scipy.org).

To interrogate the relationship between solid and fluid variables quantitatively, Fig 6 shows heatmaps of solid stress versus permeability and interstitial fluid velocity (IFV), respectively. The maps were produced using nodal data from all elements in the PTS. Strong, significant (|t| > 0.9, p < 0.001) correlations are observed in both cases: solid stress and permeability are negatively correlated, while solid stress and IFV are positively correlated. These observations are driven by the effect of microstructural remodelling: as collagen is compressed and stretched it becomes more closely packed, producing a stiffer, less permeable stroma.

## Discussion

In this paper we have presented a computational model of avascular poroelastic tumour growth coupled with a multiscale biphasic description of the host tissue. The model builds on our previous work to provide a more physiologically-representative description of the biophysical tumour-host environment, allowing us to study the interplay between solid micromechanics and peritumoural fluid flow. To promote the wider use and development of multiscale cancer models, we have also provided a primer on the mathematical formulation and implementation of the model.

Simulations were performed using experimental data to specify the model as a tumour spheroid growing into a dense, randomly organised collagen hydrogel. This produced passive mechanical remodelling of collagen fibres around the tumour periphery—recapitulating our previous findings [18] and experimental observations [4]—and revealed that this remodelling caused the periphery to become more permeable in the circumferential than radial direction. During growth, however, this effect was outweighed by large radial fluid pressure gradients. The resulting magnitudes of IFP and IFV agreed qualitatively with previous experiments ([35] and [34], respectively).

To further examine the effect of collagen orientation on IFV, simulations were compared between an initially random and a pre-aligned peritumoural collagen microstructure. Different IFV profiles were observed at the boundary, with larger circumferential components in the latter case, indicating a dependency of IFV on initial collagen orientation. This was primarily due to the pre-aligned PTS causing a heterogeneous remodelling at the boundary and subsequently a more anisotropic growth, driving up circumferential fluid pressure gradients.

Finally, the relationships between solid stress and permeability and solid stress and IFV at the tumour-host interface were investigated. Nodal data were used to establish correlations, showing that solid stress and permeability (IFV) have a negative (positive) correlation. This is intuitive: as the tissue becomes compressed the solid stress and IFV increases, while the permeability decreases. These results point to a heterogeneous environment of solid and fluid forces at the tumour-host interface, and hence the necessity for multiphase models.

The validation of the multiscale model presented here is mainly qualitative, and is restricted to the structural remodelling of the collagen networks. To provide more stringent testing of the model’s ability to predict passive structural remodelling, the change in collagen structure could be measured in vitro and in vivo using techniques such as second harmonic generation imaging [4]. To validate the prediction of increased circumferential to radial permeability at the boundary it would be necessary to either measure the hydraulic conductivity directly, or measure the interstitial fluid velocity (IFV). The latter would be easier, as the former requires model assumptions while measuring the IFV could be achieved by fluorescent labelling and subsequent imaging of suitably small particles [36]. In addition, these techniques could also be used to test the prediction that IFV is dependent upon initial collagen structure. There are a number of three-dimensional in vitro models in the literature that would be suitable for such experiments: collagen-embedded spheroid models [37], [38]; a spheroid model embedded in a compressed collagen gel, providing a more biomimetic environment [39]; and a spheroid model embedded in engineered nanofibrous scaffolds [40], that could enable the fabrication of specific microstructures.

In terms of integrating the proposed model with other computational models, a strength of the proposed method is that it is inherently modular: the microstructure is defined in a separate domain that is coupled to the macroscale space. As such it can easily be implemented in any standard finite element solver, or indeed any solver that utilises numerical quadrature e.g. [41], [42], [33], to name only a few. Furthermore, our model can be compared directly to other mathematical models that utilise similar computational methods, such as the phase-field formulation proposed by [43] which is solved using isogeometric analysis [44].

It is worth noting the limitations of the current model, particularly with a view to its application to in-vivo tumour growth and patient-specific modelling [45]. First, the peritumoural stroma is modelled as a mixture of collagen and water; in-vivo, the extracellular matrix is also comprised of other matrix proteins. This can be addressed by simply adding a constitutive description of the other matrix proteins and assuming superposition of stresses; this “two component” model was investigated in our previous work [18]. Similarly, in the current model only the peritumoural stroma is treated as a multiscale material, which is reasonable for in-vitro spheroid growth as the spheroids tend to have a low collagen content [30]; what collagen there is would be unstructured and compressed. The situation is not the same in-vivo, however, as the tumour grows from within the ECM. Again the model can be extended to accommodate this by making the tumour a two component model. Another limitation of the current model is the assumption of constant solid and fluid volume fractions, which may be expected to change in-vivo due to active remodelling by ECM cells. However, studies have shown that lymphatic vessels in the peritumoural stroma can drain fluid that leaks from the tumour [46], thus maintaining an approximately constant fluid volume fraction. The current modelling framework can be easily extended to test this by combining it with our existing model of vascular tumour growth [33].

## Supporting information

S1 File Finite element equations. Here we provide the finite element form of the coupled macro-micro conservation equations.
(PDF)

S1 Table Material parameters. This table lists the material parameters used in all simulations, unless stated otherwise in the text.
(PDF)
