# Supplementary material for: Multiscale biphasic modelling of peritumoural collagen microstructure: The effect of tumour growth on permeability and fluid flow
Source: PLoS One. 2017 Sep 13;12(9):e0184511. doi: 10.1371/journal.pone.0184511 (PMC5597211; doi:10.1371/journal.pone.0184511)
Supplement: S1 File — Here we provide the finite element form of the coupled macro-micro conservation equations. (PDF) [file pone.0184511.s001.pdf]

SUPPORTING INFORMATION

Multiscale biphasic modelling of peritumoural collagen microstructure: the effect of tumour growth on permeability and fluid flow

Peter A. Wijeratne, John H. Hipwell, David J. Hawkes, Triantafyllos Stylianopoulos, Vasileios Vavourakis

S1 File. Finite element equations. At the macroscale, the finite element form of Eq 1 is:

$$\frac{1}{(\Delta t)^2} M_{ij} \, {}^{t+\Delta t}\boldsymbol{U}_j = \frac{1}{(\Delta t)^2} M_{ij} \, {}^{t}\boldsymbol{U}_j - {}^{t}\boldsymbol{F}_i + \boldsymbol{D}_{ik} \, {}^{t}P_k + {}^{t}\boldsymbol{b}_i \tag{1}$$

Here the linear quantities are defined at time t, and:

$$M_{ij} = \rho \int_\Omega \phi_i \phi_j \, d\Omega$$
$${}^{t}\boldsymbol{F}_i = \int_\Omega \frac{\partial \phi_i}{\partial \boldsymbol{X}} \, {}^{t}\boldsymbol{S} \, d\Omega$$
$$\boldsymbol{D}_{ik} = \int_\Omega \psi_i \frac{\partial \phi_k}{\partial \boldsymbol{X}} \, d\Omega$$
$${}^{t}\boldsymbol{b}_i = \int_\Omega \phi_i \boldsymbol{Q} d\Omega + \int_{\partial\Omega} \phi_i \boldsymbol{t} \, d\Gamma$$

The equivalent for Eq 2 is:

$$\frac{1}{\Delta t} M_{ij} \, {}^{t+\Delta t}P_j = \frac{1}{\Delta t} M_{ij} \, {}^{t}P_j - \theta^S \boldsymbol{D}_{ik} \cdot {}^{t}\boldsymbol{V}_k - H_{ij} \, {}^{t}P_j + {}^{t}b_i \tag{2}$$

Where:

$$M_{ij} = \frac{1}{M} \int_\Omega \psi_i \psi_j \, d\Omega$$
$$H_{ij} = (\theta^F)^2 \int_\Omega \frac{\partial \psi_i}{\partial \boldsymbol{X}} \cdot \boldsymbol{K^c} \cdot \frac{\partial \psi_j}{\partial \boldsymbol{X}} \, J_S \, d\Omega$$
$${}^{t}b_i = \int_\Omega \psi_i Q^F \, d\Omega + {}^{t}P_j \int_{\partial\Omega} \psi_i \frac{\partial \psi_j}{\partial \boldsymbol{X}} \cdot \boldsymbol{K^c} \cdot \hat{\boldsymbol{n}} \, J_{\Gamma S} \, d\Gamma$$

Here $\boldsymbol{K^c}$ is the conductivity matrix, defined in Eq 13. At the microscale, Eq 8 is solved using the backward Euler method:

$${}^{t+\Delta t}\boldsymbol{K}^{k-1} \cdot {}^{t+\Delta t}d\boldsymbol{u}^k = {}^{t+\Delta t}\boldsymbol{b} - {}^{t+\Delta t}\boldsymbol{F}^{k-1} \tag{3}$$

Here the linear equations are defined at time $t + \Delta t$ and k denotes the Newton-Raphson step, and:

$$
{}^{t+\Delta t}\boldsymbol{K}_{ij}^{k-1} = \int_{\omega} \frac{\partial \phi_i}{\partial \boldsymbol{x}} \cdot {}^{t+\Delta t}\bar{\boldsymbol{c}}^{k-1} \cdot \frac{\partial \phi_j}{\partial \boldsymbol{x}}\, d\omega
$$

$$
{}^{t+\Delta t}\boldsymbol{b}_i = \int_{\partial \xi} \phi_i {}^{t+\Delta t}\boldsymbol{t}\, d\xi
$$

$$
{}^{t+\Delta t}\boldsymbol{F}_i = \int_{\omega} \frac{\partial \phi_i}{\partial \boldsymbol{x}} {}^{t+\Delta t}\boldsymbol{s}\, d\omega - \int_{\omega} \psi_i \frac{\partial \phi_k}{\partial \boldsymbol{x}}\, d\omega \cdot {}^{t+\Delta t}p_k
$$

This requires the tangent stiffness matrix, $\bar{\boldsymbol{c}}$, to be defined. For a one-dimensional truss and using $\bar{\boldsymbol{c}} = \frac{\partial \boldsymbol{s}}{\partial \boldsymbol{\epsilon}}$, where $\boldsymbol{\epsilon}$ is the Green-Lagrange strain tensor and $\hat{\boldsymbol{n}}$ its unit vector, we have:

$$
\bar{\boldsymbol{c}} = \frac{1}{a^f} \frac{\partial ||\boldsymbol{f}||}{\partial \epsilon} \hat{\boldsymbol{n}} \otimes \hat{\boldsymbol{n}} \otimes \hat{\boldsymbol{n}} \otimes \hat{\boldsymbol{n}}
$$
