# Supplementary material for: Multiscale biphasic modelling of peritumoural collagen microstructure: The effect of tumour growth on permeability and fluid flow
Source: PLoS One. 2017 Sep 13;12(9):e0184511. doi: 10.1371/journal.pone.0184511 (PMC5597211; doi:10.1371/journal.pone.0184511)
Supplement: S1 Table — This table lists the material parameters used in all simulations, unless stated otherwise in the text. (PDF) [file pone.0184511.s002.pdf]

SUPPORTING INFORMATION

Multiscale biphasic modelling of peritumoural collagen microstructure: the effect of tumour growth on permeability and fluid flow

Peter A. Wijeratne, John H. Hipwell, David J. Hawkes, Triantafyllos Stylianopoulos, Vasileios Vavourakis

S1 Table. Material parameters.

Table 1. Material parameters used in simulations.

Parameter	Description	Value	Source
α	Growth rate parameter	1.0	[3]
β	Growth rate parameter	12.8	[3]
γ	Growth rate parameter	0.126 day^{-1}	[3]
μ	Tumour shear modulus	1.45 kPa	[3]
κ	Tumour bulk modulus	14.01 kPa	[3]
e^f	Fibre elastic modulus	1 MPa	[5]
a^f	Fibre cross-sectional area	50 nm^2	[2]
c_0	Fibre nonlinearity parameter	1.0	[4]
θ^S	Collagen solid volume fraction	2%	Equivalent to 30 mg/ml gel ([6])
V	RVE volume	1.0	This work
L	RVE total fibre length	[15,21]	This work
k_0	Tumour permeability	2.5×10^{-10} Pa m^{-2}s^{-1}	[1]
ρ	Tissue density	1000 kg m^{-3}	Approximation
M	Biot storage coefficient	1×10^{-5} Pa	Using Fig. 1 in [7] for $\theta^S = 0.02$

References

1. Roose TR, Netti PA, Munn Y L L Boucher, Jain RK. Solid stress generated by spheroid growth estimated using a linear poroelasticity model. Microvascular Research. 2003;66:204–212.

2. Chandran PL, Stylianopoulos T, Barocas VH. Microstructure-based, multiscale modeling for the mechanical behavior of hydrated fiber networks. Multiscale Modelling and Simulation. 2008;7:22–43.

3. Wijeratne PA, Vavourakis V, Hipwell JH, Voutouri C, Papageorgis P, Stylianopoulos T, et al. Multiscale modelling of solid tumour growth: the effect of collagen micromechanics. Biomechanical Modeling in Mechanobiology. 2015;15:1079–1090.

4. Stylianopoulos T, Barocas VH. Volume-averaging theory for the study of the mechanics of collagen networks. Comput Methods Appl Mech Engrg. 2007;196:2981–2990.

5. Silver FH, Horvath I, Foran DJ. Viscoelasticity of the vessel wall: the role of collagen and elastin fibers. Crit Rev Biomed Engrg. 2001;29:279–301.

6. Ramanujan S, Pluen A, McKee TD, Brown EB, Boucher Y, Jain RK. Diffusion in convection in collagen gels: implications for transport in the tumor interstitium. Biophysical Journal. 2002;83:1650–1660.

7. Simha NK, Fedewa M, Leo PH, Lewis JL, Oegema T. A composites theory predicts the dependence of stiffness of cartilage culture tissues on collagen volume fractions. Journal of Biomechanics. 1998;32:503–509.
